# Getting to the heart of cardiovascular evolution in humans

**DOI:** 10.7554/eLife.47807

**Published:** 2019-05-28

**Authors:** Alex Pollen, Bryan J Pavlovic

**Affiliations:** 1 Department of NeurologyUniversity of California, San FranciscoSan FranciscoUnited States; 2 The Eli and Edythe Broad Center of Regeneration Medicine and Stem Cell ResearchUniversity of California, San FranciscoSan FranciscoUnited States

**Keywords:** human, chimpanzee, cardiomyocytes, hypoxia, iPSCs, Other

## Abstract

Differences in the response of cardiomyocytes to oxygen deprivation in humans and chimpanzees may explain why humans are more prone to certain heart diseases.

**Related research article** Ward MC, Gilad Y. 2019. A generally conserved response to hypoxia in iPSC-derived cardiomyocytes from humans and chimpanzees. *eLife* **8**:e42374 . doi: 10.7554/eLife.42374

Cardiovascular disease is one of the leading causes of death in both humans and captive chimpanzees. However, despite having similar heart anatomies, humans and chimpanzees are prone to different types of cardiovascular disease. In humans, a build-up of fatty materials in the arteries (a condition called atherosclerosis) can restrict blood flow to the heart, which can cause myocardial ischemia. On the other hand, great apes, including chimpanzees, are more likely to suffer heart disease from myocardial fibrosis (Lowenstine et al., 2016). This condition, which rarely leads to heart disease in humans, involves increased deposition of collagen in heart tissue (Lammey et al., 2008).

Both genetic and environmental factors could be responsible for these differences. Interestingly, cholesterol is a major risk factor for atherosclerosis in humans, yet captive chimpanzees manage to avoid this condition despite having higher cholesterol levels than humans (Varki et al., 2009). However, a lack of appropriate model systems has made it difficult to investigate how genetic differences in disease susceptibility evolved between these two species.

Recent studies have shown that cardiomyocytes (heart muscle cells derived from stem cells) recapitulate many of the features of normal human and chimpanzee hearts, although there are important differences in metabolism and maturation state (Pavlovic et al., 2018). Since it is possible to grow human and chimpanzee cardiomyocytes under common environmental conditions, this provides an opportunity to study differences in the genetics of the two systems (Gallego Romero et al., 2015). Now, in eLife, Michelle Ward and Yoav Gilad from the University of Chicago report how cardiomyocytes can be used to compare the genomic consequences of myocardial ischemia in humans and chimpanzees (Ward and Gilad, 2019).

A characteristic symptom of myocardial ischemia is oxygen deprivation, also known as hypoxia, which is caused by a reduction in the flow of blood to the heart. To simulate hypoxia, Ward and Gilad first cultured cardiomyocytes in normal oxygen levels, then exposed them to a ten-fold decrease in oxygen for six hours, before returning them to normal oxygen levels. Depriving heart tissue of oxygen typically leads to increased production of reactive oxygen species (such as peroxides) that can cause DNA damage and lipid degradation. Hypoxia induced similar effects in both human and chimpanzee cardiomyocytes, suggesting that such experiments can recapitulate the effects of myocardial ischemia.

RNA sequencing revealed that nearly one third of the genes that are expressed in cardiomyocytes respond to hypoxia. Moreover, 75% of these responded in the same way in both species, suggesting that hypoxia has a widespread and largely conserved effect on gene expression. In contrast, previous work has shown that the immune responses in humans and chimpanzees are quite different (Barreiro et al., 2010).

Next, Ward and Gilad investigated how patterns of gene expression were influenced by hypoxia within these two species. Despite the similarities revealed in the RNA sequencing experiments, several hundred genes still differed in their responses. One possible explanation for these differences is that hypoxia-related transcription factors bind to response genes to varying degrees. Consistent with this possibility, hypoxia-related factors were shown to frequently bind to, or near to, conserved response genes, but not to genes with chimpanzee-specific responses. This suggests that changes in transcription factor binding, and possibly changes to the sites they bind to, may be responsible for the several hundred genes that respond differently to hypoxia in the two species.

Another notable difference is the response of a protein called RASD1, which is upregulated during hypoxia in human cardiomyocytes but not in chimpanzee cardiomyocytes. Interestingly, this gene is also upregulated in patients with myocardial ischemia and other diseases related to atherosclerosis. RASD1 could therefore have a role in regulating hypoxia-induced tissue damage in humans.

Ward and Gilad then explored the role of genes called eQTL genes (where eQTL is short for expression quantitative trait loci). Genetic variation within these genes can explain some of the disparity in mRNA levels among individuals in a population. Intersecting eQTL genes with genetic association studies has become a popular strategy for identifying disease-risk genes (Albert and Kruglyak, 2015). However, several recent lines of evidence suggest that eQTL genes may reflect mostly neutral genetic variation and are not therefore associated with any specific diseases (Jasinska et al., 2017; Tung et al., 2015).

Ward and Gilad found that there was a significant overlap between the conserved hypoxia response genes and the genes that are upregulated in patients with myocardial ischemia. This overlap indicates that the response genes are relevant to disease: however, the overlap between these genes and eQTL genes was lower than expected (Figure 1). Therefore, identifying the genetic variants and genes that influence disease-relevant responses, such as hypoxia, is likely to be more informative than traditional baseline eQTL studies. This means that stem cell models, such as those studied by Ward and Gilad, could be used more generally to study how common variation and evolved genetic differences influence other disease-relevant responses.

**Figure 1.**
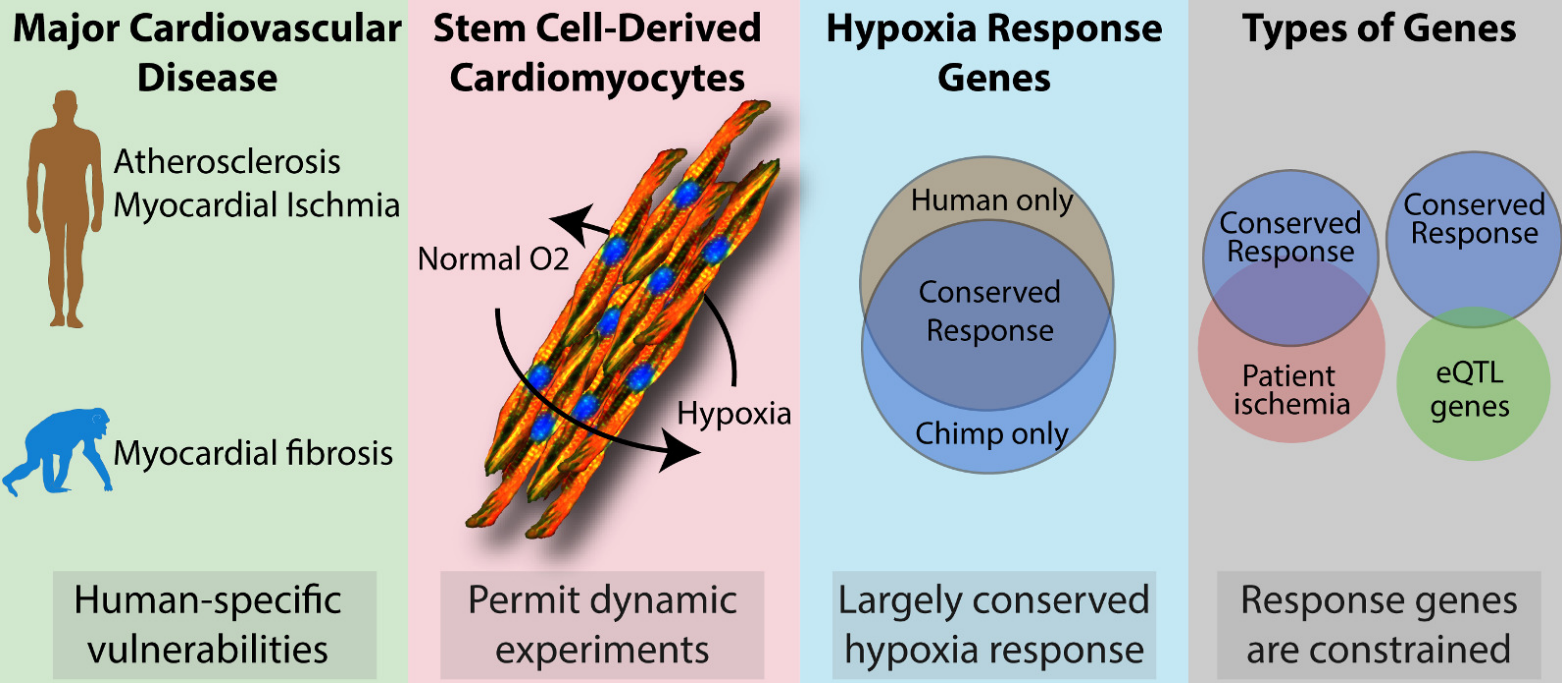
Using stem cell models to explore the genetic effects that influence susceptibility to cardiovascular disease. Ward and Gilad explored the effects of hypoxia (that is, oxygen deprivation) on cardiomyocytes (heart cells derived from stem cells) from humans and chimpanzees. They found that 75% of the genes that respond to hypoxia respond in the same way in both species (third panel). They also observed a large overlap between these conserved response genes and genes that are upregulated in patients with ischemia (fourth panel; left), but little overlap with eQTL genes (right).
